# Retraction: Neurogenic Stunned Myocardium: A Literature Review 

**DOI:** 10.7759/cureus.r12

**Published:** 2019-04-16

**Authors:** Asad Ali, Malik Qistas Ahmad, Muhammad Bilal Malik, Zara Z Alvi, Waleed Iftikhar, Deepak Kumar, Usama Nasir, Nouman Safdar Ali, Zohaib Sayyed, Risham Javaid, Neha Waqas, Shahzad Ahmed Sami, Abbas M Cheema

**Affiliations:** 1 Internal Medicine, CMH Lahore Medical College and Institute of Dentistry, Lahore, PAK; 2 Hematology/Oncology, University of Arizona Cancer Center, Tucson, USA; 3 Internal Medicine, Shifa College of Medicine, Lahore, PAK; 4 Internal Medicine, CMH, Lahore Medical College, Lahore, PAK; 5 Internal Medicine, Jinnah Postgraduate Medical Centre, Karachi, PAK; 6 Internal Medicine, Civil Hospital Sukkur, Gambat, PAK; 7 Internal Medicine, Combined Military Hospital Lahore Medical College and Institute of Dentistry, Lahore, PAK; 8 Internal Medicine, Allama Iqbal Medical College, Lahore, PAK; 9 Pediatrics, Shaikh Khalifa Bin Zayed Al-Nahyan Medical and Dental College, Lahore, PAK; 10 Medicine, Akhter Saeed Trust Hospital, Lahore, PAK; 11 Surgery, Shaikh Khalifa Bin Zayed Al Nahyan Medical & Dental College, Broken Bow, PAK; 12 Internal Medicine, Combined Military Hospital, Lahore, PAK

This article has been retracted at the request of the Editor-in-Chief as it contains significant plagiarism from Biso S, Wongrakpanich S, Agrawal A, Yadlapati S, Kishlyansky M, Figueredo V. A Review of Neurogenic Stunned Myocardium. *Cardiovasc Psychiatry Neurol*. 2017;2017:5842182. 10.1155/2017/5842182

A key condition of article submission is that authors must explicitly declare that their work is original and has not appeared in a publication elsewhere. Re-use of any data must be appropriately cited. This duplication was not detected by Cureus plagiarism check software as much of the material had been rewritten just enough to avoid detection. While the authors have maintained that this was the result of working with a contract editor, they are ultimately responsible for the draft submitted.

As such this article represents a severe abuse of the scientific publishing system. The Cureus Journal of Medical Science takes a very strong view on this matter and we apologize that this was not detected during the submission process. The relevant author institutions and departments have been notified.

